# In darkness, remember the light: *Chlamydomonas* retains low- and high-light-induced acclimatory phenotypes in the dark

**DOI:** 10.1093/plcell/koaf130

**Published:** 2025-05-23

**Authors:** Guy Levin

**Affiliations:** Assistant Features Editor, The Plant Cell, American Society of Plant Biologists; Department of Plant and Microbial Biology, University of California, Berkeley, CA 94720-3102, USA

Plants encounter a wide variety of light conditions throughout their life cycle. For example, during winter, they may experience long periods of limited light, while in summer, light may be in excess. Light availability also changes during the day, where light is limited at dawn and dusk, while plentiful during midday. Photoautotrophs' metabolism is tightly synchronized with diurnal light via multiple mechanisms, including circadian regulation and responses to external cues (Oravec and Greenham 2022; De Barros Dantas et al. 2023). In the green alga Chlamydomonas (*C. reinhardtii*), cell growth occurs during the day when light is available, while genome replication and cell division occur at the beginning of the night (Figure) (Umen and Liu 2023). Low light can lead to retarded growth due to energy limitation, but excessive light may cause a similar effect due to enhanced reactive oxygen species formation (Erickson et al. 2015). Photosynthetic organisms can respond to such changes in light availability by increasing light-harvesting under limited light and both limiting light absorption and enhancing energy dissipation under excess light (Figure) (Croce 2020). To date, it is unclear if the diurnal programming of photosynthetic metabolism in Chlamydomonas plays a role in protecting the photosynthetic machinery from excess light or if exposure to suboptimal light intensities affects the diurnal program.

**Figure.**
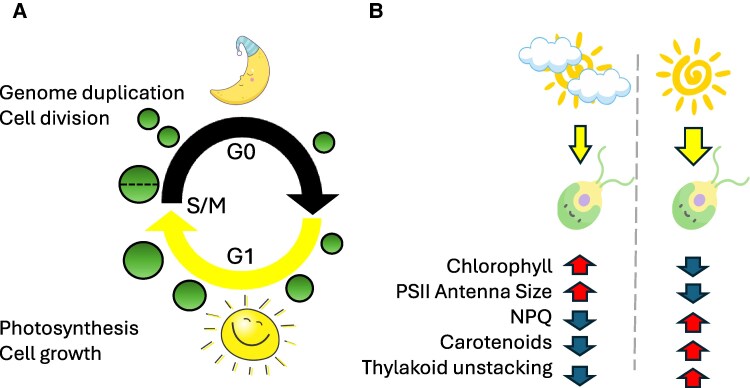
Chlamydomonas **A)** diurnal cell cycle and **B)** acclimatory response to limiting (left) or excessive (right) diurnal light conditions. Figure credit: G. Levin.

In new work, **Sunnyjoy Dupuis, Valle Ojeda, and colleagues (Dupuis et al. 2025)** use a systems approach to analyze the effect of diurnal light intensity on Chlamydomonas cells synchronized with 12-hour-light/-dark (day/night) and temperature cycles. The growth of cells under both limiting low light (LL) or excessive high light (HL) was retarded compared to cells grown under optimal, medium light (ML). However, light intensity had little impact on the Chlamydomonas cell cycle, which maintained synchronized cell division upon transition to the dark. Similarly, LL-, ML-, and HL-grown cells showed similar diurnal rhythmic gene expression programs—that is, gene and protein expression peaks simultaneously in cells grown under optimal or suboptimal light. Nevertheless, the expression of many specific genes and proteins was altered in LL- and HL-grown cells compared to ML, including enhanced accumulation of photoprotective proteins in the latter.

Interestingly, the variation in expression was not limited to the light phase but persisted after 10 h of darkness, showing that the daytime light intensity affects gene and protein expression at night. Unstacking of thylakoid membranes in HL-grown cells, which allows for efficient repair of photodamaged photosynthetic machinery, was also retained during the dark phase. The capacity to maintain high levels of nonphotochemical quenching (NPQ), where excess light is harmlessly dissipated as heat via proteins and carotenoids (Erickson et al. 2015), was also maintained in HL-grown cells during the night, with evidence suggesting lutein is the key quencher of the fastest NPQ component, q_E_. Unlike q_E_, other components of NPQ, including state transitions (q_T_) and Zeaxanthin-dependent quenching (q_Z_), were mostly active during the day but less prevalent at night. During q_T_, subunits of PSII antenna, the light-harvesting complex II (LHCII), are transferred to PSI to reduce excitation pressure from PSII. In q_Z_, zeaxanthin accumulates and provides a pathway for dissipation of excess energy (Erickson et al. 2015).

Maximal PSII efficiency was lower in HL-grown cells at the beginning of the light phase but recovered after 10 h, suggesting that Chlamydomonas adjusts and repairs its photosynthetic machinery after long exposure to diurnal HL. Higher chlorophyll a/b ratios in HL-grown cells indicated a reduction of the PSII antenna size, as chlorophyll b mainly resides in LHCII. A smaller antenna size (fewer LHCII subunits and chlorophylls) protects photosynthesis by reducing light absorption under HL conditions and was recently suggested to be a key photoprotective mechanism in the HL-tolerant alga *Chlorella ohadii* (Levin et al. 2024). The authors also noted the accumulation of the LHCII subunit LHCBM9 in HL-grown cells after 10 h of light exposure. LHCBM9 was shown to enhance the stability and energy dissipation of PSII (Grewe et al. 2014) and may be key to the recovered PSII efficiency observed after 10 h of light exposure.

This work shows that the Chlamydomonas diurnal program is robust and maintained under challenging light conditions whether limiting or in excess. Moreover, Chlamydomonas seems to retain HL-dependent acclimatory phenotypes during the night, as indicated by unstacked thylakoid membranes, reduced chlorophyll and photosynthetic protein abundance, and elevated levels of lutein, NPQ capacity, and the photoprotective q_E_-inducing protein LhcSR3 (Figure). These findings suggest that Chlamydomonas “remembers” the light intensity it encountered during the day, perhaps as a mechanism to prepare for the light levels that are expected on the following day. This complex mechanism, coordinated by a crosstalk between multiple regulatory processes, including circadian regulation and light and temperature sensing, would be beneficial for optimizing light harvesting over long periods, such as seasonal changes. In future work, it would be interesting to investigate the individual roles of each component of this mechanism, which are poorly understood in unicellular phototrophs.

## Recent related articles in *The Plant Cell*

- (Ostermeier et al. 2024) reviewed the latest knowledge of the structure, biogenesis, and evolution of thylakoid membranes, including their repair post-light-induced damage.
- (Romero-Losada et al. 2025) used a multiomics approach to determine the effect of different daylight durations on the green alga *Ostreococcus tauri*.

## Data Availability

No data was generated or analyzed during the preparation of this work.

## Dive Curated Terms

The following phenotypic, genotypic, and functional terms are of significance to the work described in this paper:

LHCBM9 Gramene: Cre06.g284200
LHCBM9 Araport: Cre06.g284200
